# Long-Term Outcomes of Pulmonary Metastasectomy for Uterine Malignancies: A Multi-institutional Study in the Current Era

**DOI:** 10.1245/s10434-020-08426-5

**Published:** 2020-04-09

**Authors:** Ryu Kanzaki, Yoshiyuki Susaki, Koji Takami, Yasunobu Funakoshi, Yasushi Sakamaki, Ken Kodama, Hideoki Yokouchi, Naoki Ikeda, Yoshihisa Kadota, Teruo Iwasaki, Naoko Ose, Yasushi Shintani, for the Thoracic Surgery Study Group of Osaka University (TSSGO)

**Affiliations:** 1 Department of General Thoracic Surgery, Osaka University Graduate School of Medicine, Suita-shi, Osaka Japan; 2 Department of General Thoracic Surgery, Osaka International Cancer Institute, Osaka, Japan; 3 Department of Thoracic Surgery, National Hospital Organization Osaka National Hospital, Osaka, Japan; 4 Department of Thoracic Surgery, Osaka General Medical Center, Osaka, Japan; 5 Department of Thoracic Surgery, Osaka Police Hospital, Osaka, Japan; 6 Department of Thoracic Surgery, Yao Municipal Hospital, Yao, Japan; 7 Department of Thoracic Surgery, Suita Municipal Hospital, Suita, Japan; 8 Department of Thoracic Surgery, Sakai City Medical Center, Sakai, Japan; 9 Department of Thoracic Surgery, Osaka Habikino Medical Center, Habikino, Japan; 10 Department of Thoracic Surgery, Japan Community Healthcare Organization (JCHO) Osaka Hospital, Osaka, Japan

## Abstract

**Background:**

Information on pulmonary metastasectomy (PM) for uterine malignancies in the current era is limited. In the present study, we analyzed the clinical course and results of PM for uterine malignancies in the era of modern imaging diagnostics to clarify the role of PM in the current era in a multi-institutional setting.

**Methods:**

Fifty-seven patients who underwent PM for uterine malignancies between 2006 and 2015 were retrospectively reviewed. The short- and long-term outcomes, along with factors associated with the prognosis, were analyzed. Details of the clinical course after PM were described.

**Results:**

The mean age of patients was 59.4 years. The primary tumor was located in the uterus corpus in 34 cases (60%) and in the uterus cervix in 23 cases (40%). The median disease-free interval (DFI) was 32 months. Forty patients (70%) received fluorine-18-2-fluoro-2-deoxy-d-glucose positron emission tomography/computed tomography before PM, and complete resection was achieved in 52 patients (91%). Postoperative complications occurred in 4 patients (7%). Of the 52 patients who underwent complete resection of pulmonary metastases, 28 experienced recurrence, and among these, 17 (60%) underwent local therapy, including six repeat PMs. Among the 52 patients who underwent complete resection, the 5-year relapse-free survival rate was 40.7% and the 5-year overall survival (OS) rate was 68.8%. The univariate analysis revealed that a DFI of ≤ 24 months was associated with significantly poorer OS.

**Conclusions:**

PM for uterine malignancies is safe and provides favorable long-term outcomes in selected patients. Patients with a DFI of > 24 months have better OS and are good candidates for PM.

Uterine malignancies are the fifth most common type of cancer and the eighth leading cause of death in women in Japan.^1^ Uterine malignancies consist of uterine corpus malignancies, which include endometrial cancer that originates from the lining of the uterus; uterine sarcoma, which originates from the muscles or support tissue of the uterus; and cervical cancer. It is reported that the rate of lung metastasis is higher in patients with sarcoma than in patients with epithelial gynecologic cancers.^2^–^4^ Because most tumors in patients with epithelial gynecologic malignancies metastasize to the pelvis, vagina, peritoneum, or lymph nodes, the incidence of isolated pulmonary metastasis (i.e. pulmonary metastasis without metastasis to other organs) is low in patients with recurrent disease.

Pulmonary metastasectomy (PM) is an established mode of treatment for various cancer types.^5^ PM is usually indicated for isolated pulmonary metastasis and there have been numerous reports on its application in the treatment of other cancers such as colorectal cancer and renal cell carcinoma;^6^–^8^ however, the information on PM for uterine malignancies is limited due to the relatively low incidence of isolated pulmonary metastasis.

Since 2006, when the use of fluorine-18-2-fluoro-2-deoxy-d-glucose positron emission tomography/computed tomography (FDG-PET/CT) in general clinical practice in Japan became widespread, there has been remarkable improvement in the ability to diagnose recurrent uterine malignancies (which directly influences the selection of patients for PM) based on medical imaging. In this clinical context, there is a need for information on the performance of PM for uterine malignancies in the current era.

In the present study, we analyzed the clinical course and results of PM for uterine malignancies in the era of modern diagnostic imaging to clarify the role of PM in the current era in a multi-institutional setting.

## Patients and Methods

In the present study, data from 10 different thoracic surgery departments belonging to the Thoracic Surgery Study Group of Osaka University were analyzed. From January 2006 to December 2015, 57 patients underwent pulmonary resection to treat pulmonary metastases originating from uterine malignancies. The study protocol was approved by the Institutional Review Boards of all participating hospitals, including that of the Ethics Committee of Osaka University Hospital (control number 17255-2). Patients who underwent surgery for the purpose of biopsy were not included in the present study. The diagnoses of the pulmonary nodules were made based on the chest CT findings or CT and FDG-PET/CT. Whether or not to perform FDG-PET/CT to determine the indication for PM is decided by the gynecologist in charge. The primary tumor was diagnosed pathologically and treated by surgery or chemoradiotherapy in all patients prior to PM. Tissues of pulmonary nodules resected were evaluated histologically and diagnosed as pulmonary metastasis from uterine malignancies were diagnosed in all cases. Clinical information was collected from the medical records in participating hospitals.

Patients generally underwent resection of pulmonary metastases after meeting the following criteria (these criteria are quoted from a previous study^9^): “(1) complete resection of the pulmonary metastasis (or metastases) was considered to be achievable; (2) the metastatic lesions were limited to the lungs, or extra-pulmonary distant metastases was already controlled or controllable if present; (3) the patient’s primary tumor was already controlled or controllable; (4) lymph node metastasis from the pulmonary lesion was determined to be absent by a preoperative evaluation; (5) the general condition of the patient was good, and the patient’s respiratory function was sufficient to tolerate pulmonary resection.”

When the patient again met the criteria for pulmonary resection, repeat pulmonary resection was also performed for recurrent pulmonary metastasis. The disease-free interval (DFI) was defined as the interval between treatment for the primary tumor and detection of pulmonary metastases, and counted as 0 for patients whose primary and metastatic disease was simultaneously diagnosed at initial presentation. The type of resection and surgical approach were selected according to the size and location of the recurrent pulmonary metastases.

The indications for pre- and/or postoperative chemotherapy were determined by the gynecologists in charge after considering the general condition of the patient and the state of the disease.

Follow-up was generally based on chest X-ray or chest CT, physical examination, and blood chemistry, which were performed every 6–12 months after the first PM. The primary endpoint was overall survival (OS), defined as the time between the date of pulmonary resection and death or last follow-up for surviving patients. Relapse-free survival (RFS) was defined as the time interval between the first PM and the first recurrence of primary tumor cancer or death due to any cause. In the present study, the follow-up period was defined as the interval between the date of pulmonary resection and the date of death or latest follow-up. In the present study, the 75th quartile, median, and 25th quartile follow-up time was 89, 46, and 27 months, respectively (range 1–133 months).

Statistical analyses were performed using the JMP Pro 13 software program (SAS Institute, Berkley, CA, USA). Descriptive statistics were reported for continuous variables, and count and percentage were reported for categorical variables. OS and RFS after pulmonary resection were analyzed using the Kaplan–Meier method. The significance of differences between subgroups was calculated using the log-rank test. *P *values < 0.05 were considered to indicate statistical significance. The data are expressed as the mean ± standard deviation or median values.

## Results

The characteristics of the 57 patients are shown in Table 1. The mean age was 59.4 years. The tumor was located in the uterus corpus in 34 patients (60%) and the uterus cervix in 23 patients (40%). Fifty patients (88%) underwent treatment, including surgery for the primary tumor. The median DFI was 32 months. Forty-one patients (72%) had a solitary pulmonary tumor, while 16 (28%) had multiple pulmonary tumors. The mean size of the pulmonary tumor was 2.0 cm. Forty patients (70%) received FDG-PET/CT before PM.

**Table 1:** Patient characteristics

Factor	
Age, years	
Mean	59.4 ± 12.3
Range	32–83
Location of the primary tumor, histologic type	
Uterus corpus, endometrial carcinoma	25 (44)
Uterus corpus, leiomyosarcoma	7 (12)
Uterus corpus, carcinosarcoma	1 (2)
Uterus corpus, adenosarcoma	1 (2)
Uterus cervix, squamous cell carcinoma	14 (25)
Uterus cervix, adenocarcinoma	8 (14)
Uterus cervix, other	1 (2)
Stage of primary tumor (FIGO classification)	
I	27 (47)
II	12 (21)
III	8 (14)
IV	3 (5)
Unknown	7 (12)
Treatment of the primary tumor	
Surgery alone	41 (72)
Surgery, adjuvant chemotherapy	4 (7)
Surgery, adjuvant chemoradiotherapy	4 (7)
Neoadjuvant chemotherapy, surgery, adjuvant chemoradiotherapy	1 (2)
Radiotherapy	2 (4)
Chemoradiotherapy	4 (7)
Unknown	1 (2)
Recurrence before pulmonary resection	
Yes	16 (28)
No	41 (72)
DFI, months (synchronous: 1 patient; unknown: 2 patients)	
75th quartile	55
Median	32
25th quartile	15
Range	0–147
Number of tumors	
Solitary	41 (72)
Multiple	16 (28)
Range	1–11
Laterality	
Unilateral	51 (89)
Bilateral	6 (11)
Size of tumor, cm	
75th quartile	2.4
Median	1.4
25th quartile	0.9
Mean	2.0 ± 1.5
Range	0.5–6.0

Data are expressed as *n* (%) unless otherwise specified

*FIGO* International Federation of Gynecology and Obstetrics, *DFI* disease-free interval

The surgical factors are shown in Table 2. Complete resection was achieved in 52 patients (91%). Wide wedge resection was the procedure most frequently performed for PM (*n* = 28, 49%). Video-assisted thoracic surgery was introduced in 38 patients (70%), and the remaining patients underwent open thoracotomy (30%). Pulmonary resection was not associated with perioperative mortality. Postoperative complications occurred in 4 patients (7%).

**Table 2:** Operation-related factors

Factor	
Preoperative chemotherapy	
Yes	11 (19)
No	45 (79)
Unknown	1 (2)
Approach	
Open	19 (33)
VATS	38 (67)
Type of resection	
Wedge	28 (49)
Segmentectomy	9 (16)
Lobectomy	19 (33)
Bilobectomy	1 (2)
Lymph node dissection	
Not done	42 (74)
Hilar	6 (11)
Mediastinal	9 (16)
Lymph node metastasis	
Yes	4 (7)
No	53 (93)
Complete resection	
Yes	52 (91)
No	5 (9)
Postoperative complications	
Yes	4 (7)
No	52 (91)
Unknown	1 (2)
Postoperative chemotherapy	
Yes	22 (39)
No	33 (58)
Unknown	2 (4)

Data are expressed as *n* (%)

*VATS* video-assisted thoracic surgery

**Fig. 1:**
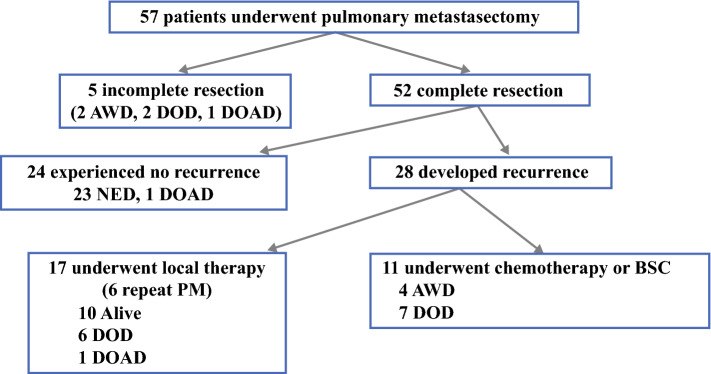
Clinical courses after the first pulmonary metastasectomy. *AWD* alive with disease, *DOAD* died of another disease, *DOD* died of disease, *NED* no evidence of disease, *PM* pulmonary metastasectomies, *BSC* breast-conserving surgery

The clinical courses of patients after the first PM are shown in Fig. 1. Of the 52 patients who underwent complete resection of pulmonary metastases, 24 experienced no recurrence and 28 patients experienced recurrence. Among the 28 patients who experienced recurrence, the initial sites of recurrence after PM were the lung (*n* = 13), the mediastinal or mediastinal and supraclavicular lymph nodes (*n* = 6), local relapse of the primary tumor (*n* = 2), and other sites (*n* = 7). Of these 28 patients, 17 (60%) underwent local therapy, including repeat PM in six cases.

**Fig. 2:**
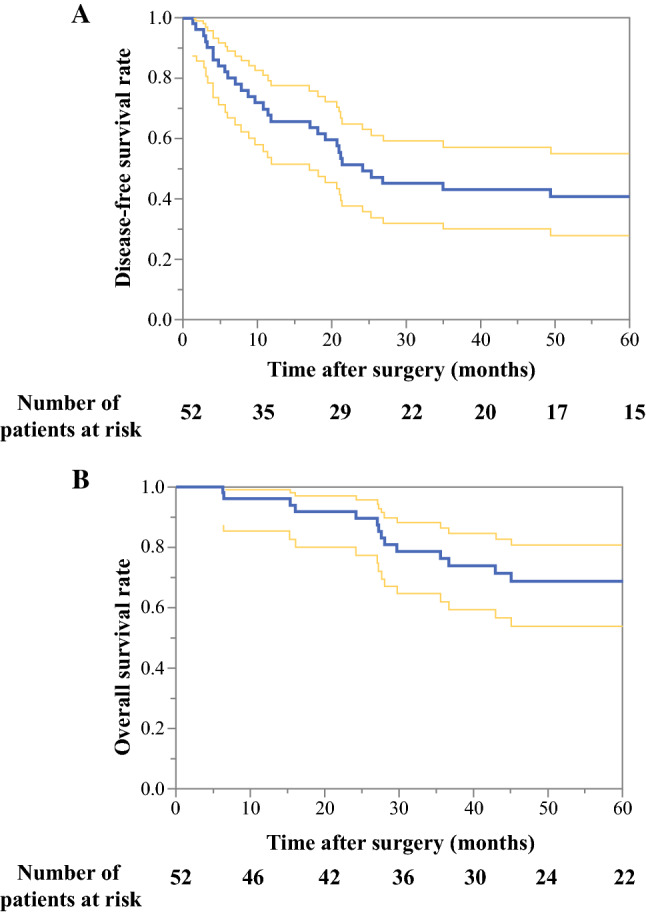
Survival analysis of 52 patients who underwent complete resection of pulmonary metastases. Dotted line indicates 95% CI

**Fig. 3:**
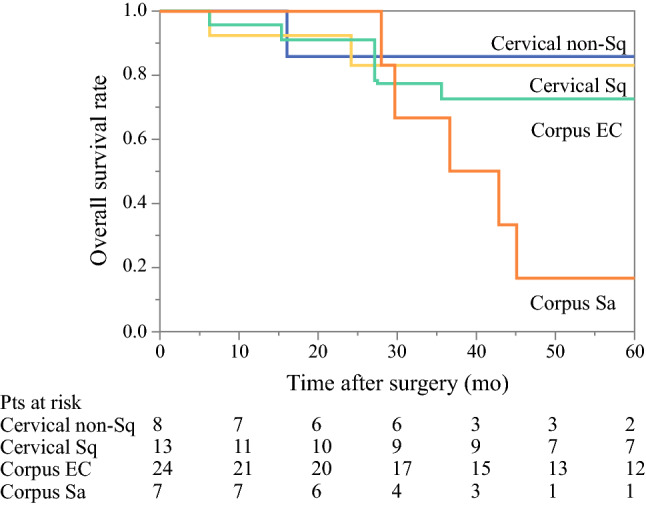
Overall survival for 52 patients who underwent complete resection of pulmonary metastases according to location and histological type. *EC* endometrial carcinoma, *Pts* patients, *Sa* sarcoma, *Sq* squamous cell carcinoma

Next, a survival analysis and an analysis of prognostic factors were conducted for 52 patients who underwent complete resection of pulmonary metastases. The 3- and 5-year RFS rates were 43.1% (95% confidence interval [CI] 30.1–57.2) and 40.7% (95% CI 27.9–55.0), respectively, while the 3- and 5-year OS rates were 76.2% (95% CI 61.9–86.4) and 68.8% (95% CI 53.7–80.6), respectively (Fig. 2). OS according to location of the histologic type is shown in Fig. 3. Patients with sarcomas in the uterus corpus had the worst outcome, however the number of patients was small. The 5-year OS rates of patients with cervical non-squamous cell carcinoma, cervical squamous cell carcinoma, corpus endometrial carcinoma, and corpus sarcoma were 85.7%, 83.1%, 72.6%, and 16.7%, respectively.

Univariate analysis of factors associated with survival in 52 patients who underwent complete resection of pulmonary metastases was conducted using the following factors: age, location of the primary tumor, stage of the primary tumor, recurrence before pulmonary resection, DFI, number of resected tumors, tumor size, type of resection, lymph node involvement, and postoperative chemotherapy. The results of the univariate analysis are shown in Table 3. The location and size of the primary tumor were significantly associated with RFS. On the other hand, DFI was significantly associated with OS.

**Table 3:** Univariate analyses of the factors associated with relapse-free and overall survival after pulmonary metastasectomy in patients who underwent complete resection pulmonary metastases (*n* = 52)

Factor	5-year RFS	*P* value	5-year OS	*P *value
Age, years (≤ 60 vs. > 60)	37.0% versus 44.1%	0.37	65.4% versus 70.0%	0.88
Location of the primary tumor (uterus corpus vs. cervix)	26.7% versus 66.9%	0.01	60.0% versus 83.8%	0.13
Stage of primary tumor (I vs. II, III, IV)^a^	46.2% versus 37.5%	0.61	74.3% versus 69.6%	0.40
Recurrence before pulmonary resection (yes vs. no)	42.9% versus 39.5%	0.78	76.6% versus 66.2%	0.53
DFI (≤ 24 months vs. > 24 months)^a^	33.3% versus 46.4%	0.17	51.9% versus 81.5%	0.04
Number of tumors (solitary vs. multiple)	44.2% versus 30.8%	0.36	68.9% versus 67.1%	0.60
Size of tumor (≤ 2 cm vs. > 2 cm)	48.8% versus 10.6%	< 0.01	75.1% versus 40.0%	0.06
Type of resection (sublobar vs. lobectomy)	45.5% versus 31.7%	0.41	66.5% versus 74.0%	0.65
Lymph node involvement (no vs. yes)	43.7% versus 0%	0.24	68.7% versus 66.7%	0.80
Postoperative chemotherapy (yes vs. no)^a^	32.4% versus 47.1%	0.5	76.2% versus 67.5%	0.97

*DFI* disease-free interval, *OS* overall survival, *RFS* relapse-free survival

^a^Unknown cases were excluded

## Discussion

The present study demonstrated that PM for uterine malignancies provides favorable long-term outcomes in selected patients. DFI, defined as the interval between treatment for a primary tumor and detection of pulmonary metastases, is associated with OS.

Previous reports on PM for uterine malignancies from 2001 in the English literature are shown in Table 4.^10^–^17^ The numbers of patients ranged from 13 to 133, and the 5-year OS rates ranged from 47 to 82%. In the present study and three previous studies, DFI was shown to have a significant influence on long-term survival.^11^,^13^,^15^,^17^ This measure might represent the indolent or aggressive nature of a primary malignancy. Among the previous studies, the report from Anraku et al. had the largest patient population.^11^ According to the location and histological type, Anraku et al. reported that endometrial adenocarcinoma had a better outcome than cervical cancer. In their cohort, the 5-year OS rate was 75.7% in patients with endometrial adenocarcinoma, 46.8% in patients with cervical squamous cell carcinoma, 40.3% in patients with cervical adenocarcinoma, and 37.9% in patients with leiomyosarcoma. Clavero et al.^13^ also reported that among the primary locations of gynecologic cancers, cervical cancer was associated with the worst outcomes. On the other hand, cervical cancer had better outcomes in the present study; cervical non-squamous cell carcinoma showed the best outcome, followed by cervical squamous cell carcinoma, corpus endometrial carcinoma, and corpus sarcoma.

**Table 4:** Previous reports on pulmonary metastasectomy for uterine malignancies

Author	Year	Study period	Total no. of patients	Corpus Ca	Corpus Sa	Cervix	5-year OS (%)(95% CI)	Factors associated with worse prognosis
Anderson et al.^10^	2001	1982–1999	25	6	13	6	NA	NA
Anraku et al.^11^	2004	1984–2002	133	46	11	76	55 (46–64)	(Multivariate) DFI < 12 months
Yamamoto et al.^12^	2004	1983–1997	29	0	0	29	NA	(Multivariate) tumor no. ≥ 3; histology: non-Sq
Clavero et al.^13^^a^	2006	1985–2001	70	20	40	7	47 (34–63)	(Univariate) DFI < 24 months; primary site: cervix, adjuvant chemotherapy after the initial gynecologic cancer operation
Paramanathan and Wright^14^	2013	2001–2011	13	0	13	0	66 (NA)	NA
Adachi et al.^15^^a^	2015	1985–2013	23	4	0	14	82 (NA)	(Univariate) DFI < 24 months
Paik et al.^16^	2015	1995–2011	29	21	8	0	48 (NA)	(Univariate) symptomatic, tumor no. ≥ 3
Anile et al.^17^	2017	1997–2010	19	12	4	3	41 (NA)	(Multivariate) DFI < 24 months, recurrence after PM
Present study	2020	2006–2015	57	25	9	23	69 (54–81)	(Univariate) DFI ≤ 24 months

*Ca* carcinoma, *CI* confidence interval, *DFI* disease-free interval, *NA* not available, *PM* pulmonary metastasectomy, *OS* overall survival, *Sa* sarcoma, *Sq* squamous cell carcinoma

^a^Includes gynecologic malignancies other than uterine malignancies

In comparison with the reports by Anraku et al. (study period 1984–2002) and Clavero et al. (study period 1985–2001), the long-term outcome of PM for cervical cancer seems to have improved in the present study (study period 2006–2015), although the differences were not statistically significant. The reason for this improvement is partly explained by the introduction of FDG-PET/CT for restaging for recurrent cervical cancer. In fact, in the present study, 40 patients (70%) underwent FDG-PET/CT before PM. It is reported that the early detection of recurrent cervical cancer is correlated with improved survival.^18^ Kitajima et al.^19^ showed that FDG-PET/CT had higher diagnostic accuracy than PET or CT alone in detecting tumor recurrence. In addition, Chung et al.^20^ reported that the use of FDG-PET/CT was associated with changes in patient management in 23.1% of 52 cervical cancer patients with suspected tumor recurrence. Based on these data, it is considered that FDG-PET/CT facilitates more appropriate patient selection for PM compared with other conventional imaging modalities, and eventually results in favorable outcomes of PM.

Sarcoma in the uterus corpus had the poorest outcome in both the study by Anraku et al.^11^ and our study. Lusby et al. reported that pulmonary metastasis was one of the worse prognostic factors in the analysis of 192 patients with metastatic leiomyosarcoma.^21^ One noteworthy aspect of their report is that among patients with distant metastases, all patients who achieved 10-year survival underwent surgical resection with curative intent, with disease limited to a single organ at presentation, most commonly the lung. In line with their report, the long-term survivors (RFS of 100 months) in the present study had solitary pulmonary metastasis without other organ metastasis. In addition, two patients with other organ metastasis experienced relapse after PM. Based on these results, thoracic surgeons should carefully consider the indications for PM in patients with pulmonary metastasis from uterine corpus sarcoma who have multiple pulmonary metastases or other organ metastasis.

In the present study, the 5-year OS and RFS rates in patients with uterine malignancies who underwent complete resection of pulmonary metastases after PM were 68.8% and 40.7%, respectively. This discrepancy between RFS and OS after PM means that prolonged survival from the time of relapse after pulmonary resection was achieved. Recently, the favorable outcomes of curative intent stereotactic body radiation therapy (SBRT) as salvage therapy for recurrent gynecologic malignancies were reported.^22^ It is considered that successful local therapy with curative intent partly contributes to prolonged survival after relapse. In fact, of the 28 patients who experienced recurrence after PM, 17 (60%) underwent local therapy. To the best of our knowledge, details of the clinical course after PM for uterine malignancies have not been well reported. In the present study, we demonstrated that a high percentage of patients underwent local therapy.

It remains debatable whether PM represents a better treatment option in comparison with systemic therapy and SBRT. Recently, SBRT came to be widely performed for pulmonary metastasis from various types of cancers, which provides a favorable local control rate.^23^ The practice of PM lacks firm evidence, To date, no randomized trials have been conducted comparing PM and other treatment options.^24^ Indeed, we have no data on patients with pulmonary metastasis who do not undergo PM, thus a comparison between surgical and non-surgical patients could not be performed in the present study. The results of this study should therefore be interpreted with caution, as we demonstrated that PM seems to be beneficial for highly selected patents with pulmonary metastasis from uterine malignancies. A future study is needed to clarify whether PM is the best treatment mode for these patients.

The present study was associated with several limitations. First, the relatively small sample size limited the power of our statistical findings. Second, there may be differences in patient selection among the institutions. From the thoracic surgeon’s point of view, the indications for PM were consistent with those shown in the Methods section, however there may have been selection bias prior to referral to the thoracic surgery departments, as the doctor in charge who treated the primary tumor made the judgment. Third, we could not evaluate the direct influence of FDG-PET/CT on the outcomes due to the retrospective study design.

## Conclusion

PM for uterine malignancies is safe and provides favorable long-term outcomes in selected patients. Patients with a DFI of > 24 months have better OS and are good candidates for PM.
